# Non-Destructive Neutron Tomography Analysis of Ceramic Vessels from the Shubarat-1 Archeological Site

**DOI:** 10.3390/jimaging12070314

**Published:** 2026-07-10

**Authors:** Kuanysh Nazarov, Veronica Smirnova, Murat Kenessarin, Yekaterina Dubyagina, Yeldos Kariyev, Sergey Kichanov, Ayazhan Zhomartova, Bagdaulet Mukhametuly, Elmira Myrzabekova

**Affiliations:** 1 Institute of Nuclear Physics, Almaty 050032, Kazakhstanzhomartova@jinr.ru (A.Z.);; 2 Al-Farabi Kazakh National University, Almaty 050040, Kazakhstan; 3 Joint Institute for Nuclear Research, Dubna 141980, Russia; 4 Margulan Institute of Archaeology, Almaty 050000, Kazakhstan; 5 Suleyman Demirel University, Almaty 040900, Kazakhstan

**Keywords:** neutron tomography, Shubarat-1 archeological site, non-destructive analysis

## Abstract

We studied the structural features in internal pores and mineral inclusions of several vessels from the Shubarat-1 archeological site in the Republic of Kazakhstan, dating to the late first millennium BC, using neutron tomography. Differences in the neutron attenuation coefficients of the constituent elements of pottery objects, as well as the high penetration capability of neutron tomography, make it possible to conduct non-destructive studies of rare ceramic vessels. By analyzing the three-dimensional tomography data, we can reconstruct the size and morphological parameters of internal pores and minerals. Based on these structural findings, we can clarify past pottery production processes.

## 1. Introduction

The Shubarat-1 burial ground [1,2,3] is located near the village of Shamalgan, Karasay District, Almaty Region, Republic of Kazakhstan. The complex is associated with the Saka and Saka–Wusun periods (the late first millennium BC) and archeological investigations have periodically been undertaken there from the end of the twentieth century until recently [1,2]. Mortuary offerings included weapons and military equipment [4,5], including iron cleavers, daggers, bone arrowheads, bronze belt buckles and quiver hooks. Radiocarbon dating and ceramic seriation analysis confirm that the complex was constructed during the late Saka period (second half of the sixth century to the first half of the second century BC). Interestingly, the funerary objects have analogies in synchronous nomadic cultures from across Central Asia, including cultures of the Scythian type in the Altai-Sayan region [3,5]. Traditionally, archeologists have linked the formation of the Saka cultural repertoire to peoples in the Altai, Tuva and Western Mongolia.

In response to urban sprawl, in 2023 and 2024 [1], archeological excavations were conducted at several burial mounds at Shubarat-1. During these excavations, artifacts were recovered, including ceramic bowls and pots, AND jug-shaped containers, as well as various metal objects (knives, earrings), animal bones, and human remains. The excavated ceramic vessels [6] are characterized by a simple shape, which lacks decorative ornamentation, and often show clear traces of household use and burning. Of particular interest is an assemblage of undamaged conical–ovoid vessels, about 15–16 cm in height. Given their archeological significance, these items are difficult to study using conventional physical methods, given the destructive nature of most methods. However, nuclear–physical neutron techniques for non-destructive structural analysis [7,8,9,10,11,12], such as neutron tomography and neutron diffraction, are increasingly being implemented in other parts of the world. These nuclear physics methods ensure that the studied objects are not damaged while still providing the most comprehensive analysis of the chemical composition, internal defects, structural features of the pore space, and the spatial distribution of mineral phases within the volume of an archeological artifact [11,12,13,14]. Systematic neutron studies provide informative historical data on ceramics, including information about the methods used for pottery production, the reconstruction of ancient trade routes, and the cultural and social development of historical communities [15,16]. The experimental facility TITAN for neutron radiography and tomography has been successfully operating at the WWR-K research nuclear reactor of the Institute of Nuclear Physics in Almaty, Republic of Kazakhstan [17]. The maximum beam dimension for neutron experiments is 20 cm, which has already proven to be sufficient to examine ceramic vessels.

A quantitative analysis of internal structural characteristics and the study of spatial and morphological distributions of minerals and pores in ceramics can open new horizons for understanding pottery production processes and the sourcing of raw materials [15,18,19]. In order to produce a systematization and typology of ceramic fragments, there needs to be considerable diversity of archeological ceramic materials, with pronounced variability in the mineral or chemical composition of the clay and additives [11,12,15]. The presence or absence of pores, a quantitative analysis of pore volumes, and the morphology of open and closed pores can provide structural markers for a comparative analysis of different ceramic objects from different archeological sites. One proof-of-concept study includes neutron studies of fragments of Byzantine ceramics [15] excavated in the Dobrudja region of Romania, whereas structural data on pores and mineral inclusions have been shown to be useful for grouping ceramic items and providing possibilities for determining the location of pottery production sites based on their structural data. Tomographic studies of model ceramic tiles [19] have shown that the relative content of clay and quartz minerals is determined by differences in the volume fraction and morphology of large internal pores. Thus, neutron tomography studies of ancient ceramic vessels from the Akterek burial site [18] and the results of a detailed analysis [19,20,21] of three-dimensional data on the structural features of internal pores and minerals allow us to hypothesize about the features of thermal firing of these pottery products.

Neutron tomography can be used to contrast archeological ceramic materials from the same or different historical periods. From this perspective, the ceramic vessels discovered at Shubarat-1 provide an excellent model assemblage for studying internal inhomogeneities, such as pores, cracks and mineral inclusions. Most importantly, archeological ceramic items from the Saka period are rare pieces of cultural heritage and merit careful curation and preservation. In this study, we hope to begin the process of building a baseline of quantitative structural data for the comparative analysis of ceramic materials from historical communities across the modern territory of Kazakhstan. Here, we present the results of a study of the internal structural organization of pores and mineral inclusions in large ceramic vessels from the Shubarat-1 burial ground using the non-destructive neutron tomography method.

## 2. Materials and Methods

### 2.1. Samples Description

**Figure 1:**
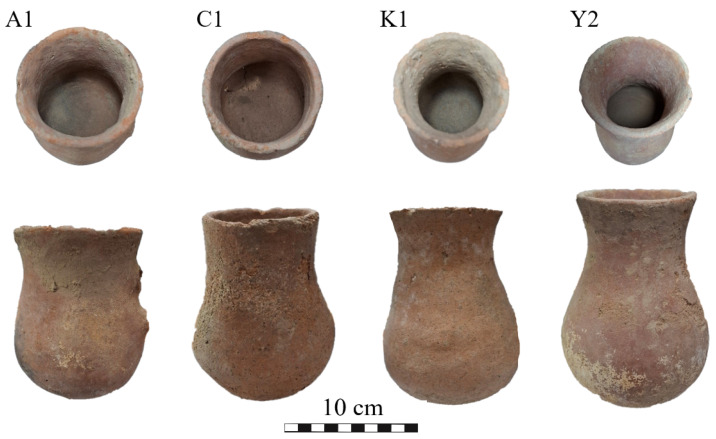
Lateral and top views of the studied vessels from Shubarat-1. The labeling of the vessels is explained in the text.

As a result of archeological work [1] in 2023–2024, fourteen funerary mounds were studied. Four unbroken vessels with a high degree of preservation were selected. Photographs of the studied vessels, each of which originated from a different burial, are presented in Figure 1. Vessel A1 was obtained from mound A1 and is an elongated pot with an expanded body and traces of a broken handle. Its height is h = 15.5 cm, and its diameter measurements were: (1) at the rim—10 cm; (2) at the neck—9.1 cm; (3) of the expanding part—11.5 cm; and of the base—9.1 cm. The reddish color of this vessel resulted from the fact that one side was heavily fired and the clay used is of medium hardness. Vessel C1, from mound C1, is almost jug-shaped, with a long neck (6.2 cm in length) that transitions smoothly into a rounded body. The base is rounded and slightly tilted to one side; the rim is straight and flattened. The vessel measurements are as follows: height 16.5 cm; rim diameter 8.7 cm; neck diameter 9 cm; body length 11 cm; and base length 8.2 cm. The surface is rough and brick-colored, and traces of smoothing with a wooden knife are visible on the interior surface. The clay matrix is crumbly and contains traces of inclusions. Vessel K1, from mound K1, is conical–oval in shape, with a moderately rounded body and a smooth transition to the neck. The neck is slightly concave, the rim is curved outward, the edge is rounded, and the base is also rounded and stable. The dimensions of this pot include a height of 16.5 cm and a diameter at the rim of 9 cm, at the neck of 7.8 cm, at the flared part of 11.2 cm, and at the base of 9 cm. The surface has been smoothed, possibly with a wooden knife, and the color is light brown. The clay is hard, with several visible inclusions tentatively identified as carbonates and feldspars. Y2 is a pear-shaped vessel and was found in mound Y2. It has a rounded body, widening toward the bottom and tapering smoothly toward the neck. The rim is straight, the edge is rounded, and the base is rounded, with the vessel leaning slightly to one side. The vessel’s dimensions are as follows: height—18 cm, neck diameter—7.8 cm, rim diameter—8 cm, base diameter—approximately 11.2 cm, and flared diameter—12 cm. The clay is medium-hard, with a smooth-grained fracture. The clay is sandy and contains inclusions tentatively identified as mica and feldspar, and it appears to have undergone a short firing period. The surface is coated with a burgundy-colored engobe, and there are also smudges on the inside from the engobe coating.

**Figure 2:**
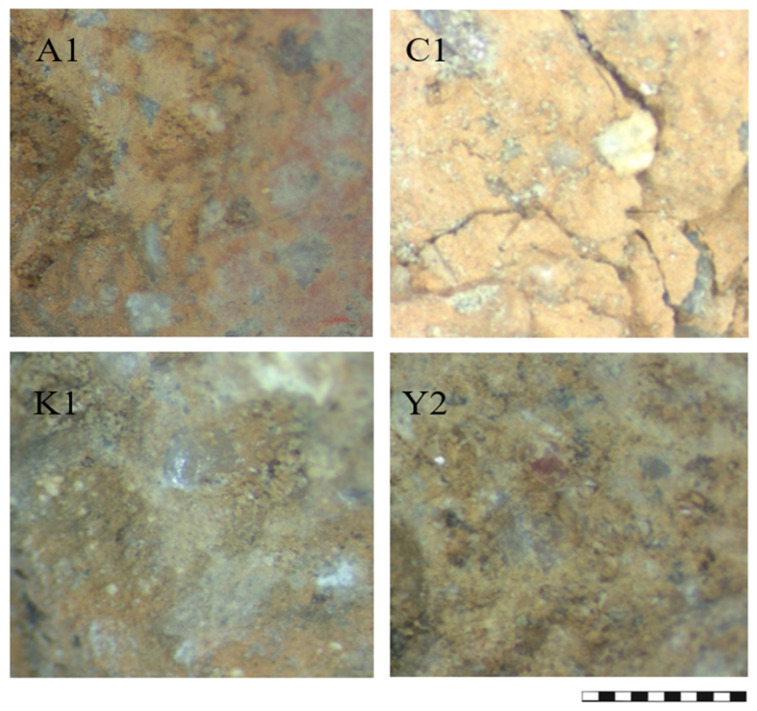
Several examples of images of vessel surfaces obtained using optical microscopy with a magnification of 3.2. The microscope scale shown corresponds to 0.2 mm.

Preliminary visual studies (Figure 2) were conducted using a Discovery V8 stereo microscope (ZEISS, Oberkochen, Germany). All vessel surfaces exhibited yellow-brown clay matrices with well-defined mineral inclusions. For sample A1, these inclusions included a tentatively small inclusion of quartz. Sample C1 has several small mineral inclusions, with an average size of 100 microns; they appear to be poorly sieved or coarse sand. Several cracks and cavities are visible on the surface of vessel C1. The surfaces of vessels K1 and Y2 have a darker color due to the different firing processes used during pottery preparation, resulting from oxidative-reductive conditions. The burgundy-colored engobe should be taken into account. Larger mineral inclusions up to a millimeter in size are visible on the surfaces of these vessels.

### 2.2. Neutron Tomography

Neutron radiography allows us to obtain a neutron image of the internal structure of various objects [16,22,23]. The image obtained by this method consists of a set of pixels that represent the degree of attenuation of the neutron beam intensity at a specific point in the object being studied. While X-ray radiation has a smooth dependence on atomic number, neutron radiation has an irregular one. This difference stems from the different nature of interactions between neutrons and matter: X-rays interact with electron shells, while neutrons interact with nuclei. The technique of neutron radiography and tomography has been employed to investigate structural heterogeneities at the micron scale. In comparison to X-rays, neutron radiation exhibits a deeper penetration capacity into the material under examination, making it suitable for investigating relatively large objects with diverse shapes and dimensions.

Neutron radiography entails acquiring neutron images of the samples being analyzed, where variations in neutron absorption cross-sections among different elements provide insights into the internal distribution of heterogeneities within the materials under investigation [23]. A particular manifestation of this technique is neutron tomography, which entails reconstructing a three-dimensional (3D) representation of the sample from a series of individual radiographs obtained from various angular orientations of the sample with respect to the direction of the neutron beam. The technique of neutron tomography enables the generation of 3D models, which are composed of voxels—three-dimensional data elements with spatial coordinates and a grayscale value indicating the degree of attenuation of the neutron beam at a specific point of the analyzed sample [23].

The virtual three-dimensional representation of the studied sample, generated through tomographic reconstruction based on individual neutron projections, provides a comprehensive visualization of its intricate internal structure.

The neutron tomography experiments were performed using the TITAN facility [17,24] at the WWR-K research reactor in Almaty, Kazakhstan. A neutron beam with dimensions of 20 × 20 cm^2^ was formed by a collimator system, with the characteristic parameter L/D being equal to 350. Here, L is the distance between the input aperture of the collimator system and the position of the sample, and D is the diameter of the input aperture of the collimator system. The integral flux in thermal neutrons at the sample position was 7.2(2) × 10^6^ neutrons/(cm^2^·s). The neutron radiography data were collected using a scintillator-based detector system equipped with a high-resolution CCD camera that is based on the HAMAMATSU-S121 chip of the photo elements 24.5 mm × 24.5 mm at 2048 × 2048 pixels; each active pixel size is 12 μm × 12 μm and an LiF/ZnS:Cu scintillation screen with a thickness of 100 μm was used, manufactured by RC TRITEC Ltd. (Teufen, Switzerland). Due to the size of the studied samples, the field of view of the detector was focused, using a lens, on an area of 18.8 × 18.8 cm^2^. For the tomography reconstruction process, 360 neutron radiography images were used, each corresponding to a different angular position of the sample with respect to the neutron beam direction. Tomography experiments were performed using a goniometer with an angular rotation step of 0.5°. The exposure time for each projection was 20 s, and the total measurement time lasted 4 h. The imaging data was corrected for dark current and normalized to the image of the incident neutron beam using ImageJ 1.54 software [25]. The reconstruction of three-dimensional (3D) data from neutron projection data was carried out using the SYRMEP 1.6.3 software [26]. Iterative reconstruction algorithms for tomography have proven to be effective in producing high-quality 3D data. We executed 150 iterations of the SIRT algorithm on neutron projection data. The use of graphics cards with CUDA technology support allowed us to significantly reduce the computation time. The resulting 3D volumetric data of voxels is essential for understanding the spatial distribution of neutron attenuation coefficients within the entire vessel volume. The resulting volume of each voxel was 1.25 × 10^−4^ mm^3^. 

For analyzing porous materials, such as pottery and ceramics, using 3D neutron tomography data, a pore segmentation approach [19,20] is crucial. The local threshold approach from the watershed method was used for pore segmentation. Numerous methods and variations in segmentation techniques are employed for image analysis, and the quality of pore segmentation in the reconstructed 3D data is significantly impacted by blurring and artifacts in neutron tomography data [20,27]. To address this issue, we used a binary image indicating the approximate location of pores on the original image, which can be represented by a set of markers at the pore locations [19]. The concept behind this method is to determine a local threshold for each segmented pore using the watershed algorithm. In this neutron tomography study, we implemented additional steps in the standard watershed method. These steps involve marking the pores that were segmented using the standard watershed algorithm and calculating the threshold as the minimum value of the original image on the corresponding watershed line, or simply on the boundary of the pore. This process has been described in more detail previously [19,20]. No independent validation was performed on these specific datasets. 

The VGStudio MAX 2.2 software package, developed by Volume Graphics (Heidelberg, Germany), was used to visualize and analyze the three-dimensional data obtained.

## 3. Results and Discussions

**Figure 3:**
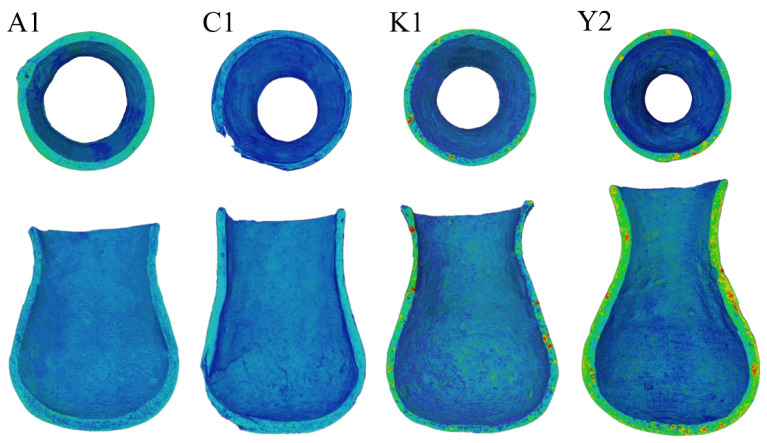
The longitudinal and transversal slices of the reconstructed 3D models of the vessels. The rainbow-like coloration indicates the degree of neutron absorption from low (green) to high (red). Clay shows up as turquoise, pore spaces are indicated in dark blue, and inclusions are indicated in yellow-red.

The 3D models of ceramic vessels were obtained using the neutron tomography reconstruction procedure (Figure 3). Based on neutron tomography data, it was possible to clearly distinguish the inner structural features of the studied vessels. The clay material itself appears to be quite uniform; however, all the vessels proved to be highly porous, and inclusions of mineral inclusions were also present in vessels K1 and Y2. We tentatively assume that the pores may be due to the presence of gases inside the ceramic matrix during the annealing process, which would have been possible if chamotte or other organic additives were present in the clay material [11,12,18]. The presence of pores in all four vessels could tentatively indicate a single source of clay, as well as similar manufacturing procedures and annealing conditions.

The corresponding internal pore volumes were segmented from the entire volumes of the 3D models for statistical analysis. Both small and large pores were present in all four vessels, and their dimensions and morphological features were obtained. The volumes of vessel material, the pore volumes, and the calculated porosity of pottery objects are presented in Table 1. The porosity of the samples varies from a minimum of 0.2% for sample Y2 to a maximum of 0.78% These values represent only resolvable pore volume fraction (>~0.1 mm), not total porosity. Furthermore, samples K1 and Y2 contain additional inclusions characterized by a high neutron attenuation coefficient, with volumes of 1.22% and 1.68%, respectively, which may be suggestive of carbonate and feldspar inclusions; however, a confirmatory analysis was not performed.

**Figure 4:**
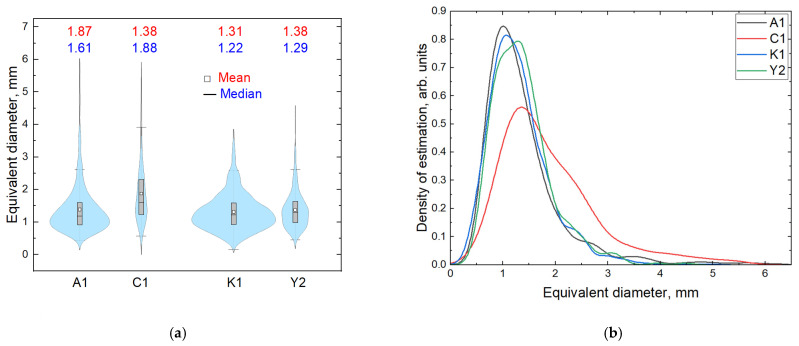

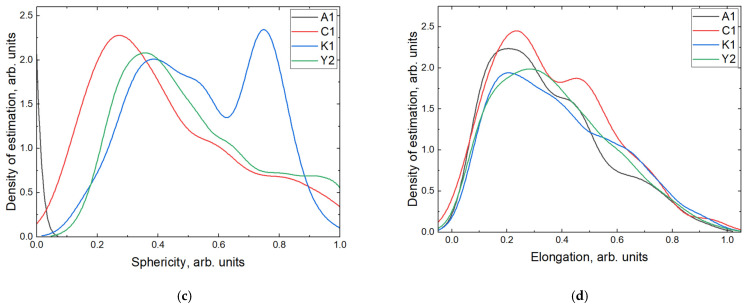
(**a**) The violin-type plot shows the probability density distribution of equivalent pore diameter is presented. Calculated values for average and median pore size are shown at the top. Normal distribution approximations for equivalent diameter (**b**), sphericity (**c**) and elongation (**d**) distributions for pores in the vessels are also presented.

For a comparative analysis of the segmented pores of the four vessels, we calculated the distributions of equivalent pore diameters [28]. The equivalent diameter is the diameter of a sphere that has the same volume as the segmented pore [29]. Then, we used a non-parametric approach to approximate the experimental data with a probability density function. We employed a kernel density estimation method with the Silverman bandwidth [30]. Within this framework, we were able to compare the calculated distributions between ceramic samples. The resulting approximation curves are presented in Figure 4a. While samples A1, K1, and Y2 have a narrow dimensional distribution within the range from 0.5 to 2.0 mm, sample C1 has a wide distribution with a prominent peak at ~2.5 mm. This indicates the presence of large pores, along relatively small pores that are characteristic of all vessels. We tentatively assume the presence of large pores, internal cracks, or cavities fused together. Together with the uneven distribution of pores and wide distribution of equivalent radius, this indicates some deviations in the technological processes used to prepare vessel C1, or may indicate a poor quality of the initial clay mixture. The absence of large mineral additives in vessels A1 and C1 may indicate a later production period for these vessels. Tellingly, the average pore size for all the vessels studied does not vary much (Figure 4b). For vessels C1, K1, and Y2, the average equivalent pore radius is about 1.3(6) mm, and for vessel A1, this parameter increases to 1.8(7) mm (Figure 4a).

To evaluate the shape of the pores, we calculated the distribution of the sphericity parameter [31]. Sphericity is the ratio of the surface area of a sphere with the same volume as a pore to the surface area of that pore. This parameter ranges from 1.0, for perfectly spherical pores, to 0.0, for particles with irregular shapes. According to Figure 4c, the median value of sphericity is approximately 0.25. This indicates that pores strongly deviate from a spherical shape and are more elongated or flattened. However, in the distribution of sphericities, there is a second peak for sample K1 at 0.75 and a smaller peak for A1 at 0.8. Such a complex distribution may indicate multiple sets of pores within vessel volumes. We assume that there are two main types of pores: elongated and spherical. Spherical pores are formed during the firing of a vessel, when gas bubbles form, while elongated pores can serve as an indicator of the production process [14,15]. We calculated the parameter of pore elongation (Figure 4d). In morphological analysis, the elongation parameter [32] quantifies how much the shape of a pore deviates from an equilateral form. The average elongation of the observed segmented pores is similar for all vessels: 0.35 for the A1 sample, 0.37 for C1, 0.39 for K1, and 0.37 for the Y2 vessel. Thus, the ratio of the principal axes of the ellipsoids is close to 3:1. Based on the calculated data, we can assume most pores have a “bladed” shape [28], which is simply described as elongated particles flattened along one of the ellipsoidal axes. This may indicate the formation of elongated pores during pottery production. Several pores show high elongation values that may be attributed to internal cracks and voids. However, there are very few of these.

A large number of mineral inclusions were found in vessels K1 and Y2 (Figure 3). According to preliminary analyses, we tentatively assume that these are inclusions of mica or quartz [11,12,13]. We further suggest that quartz or mica particles are linked to the original sand or rock inclusions. It has previously been shown [13,14] that, in Byzantine ceramics, the addition of larger shell inclusions and coarser processing of ceramic vessels are associated with different locations of clay workshops and different functional purposes for the ceramic vessel. In the case of our studied vessels, we believe that there was only one source for the clay, and the additives of mineral inclusions may have been associated with its different functional purposes. We hypothesize that vessels A1 and C1 may have served domestic functions, as suggested by the presence of soot traces, while vessels K1 and Y2 may have had different purposes. However, this interpretation remains tentative, and functional assignment requires further residue or use-wear analysis.

**Figure 5:**
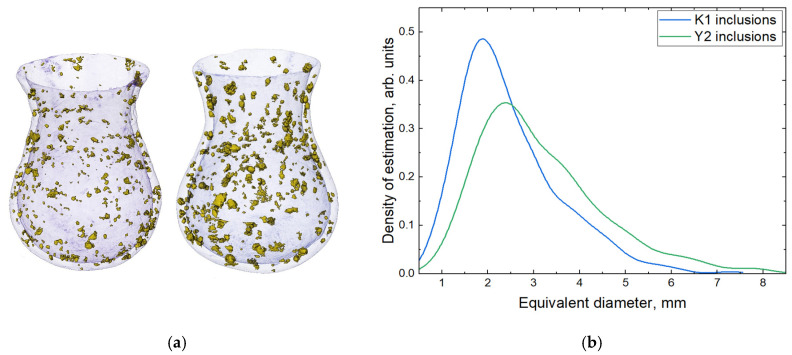
(**a**) The 3D models reconstructed from neutron tomography data of the spatial distribution of inclusions inside vessels K1 and Y2. These inclusions are marked in yellow. (**b**) The probability density functions of the distribution of the equivalent diameters for observed inclusions.

We segmented the inclusions of mineral inclusions from volumes of vessels K1 and Y2 and obtained the distribution of equivalent diameters of mineral inclusions (Figure 5). It can be seen that the dimensional distributions that were obtained do not differ greatly; however, for vessel Y2, there is a shift in the distribution towards larger sizes of mineral inclusions. However, we assume that this is due to the presence of several large inclusions or their aggregates. The average size of these mineral inclusions for vessel K1 is 3.5 mm, and for vessel Y2 it is 3.3 mm. This may indicate a similar manufacturing process was used for these pottery items.

## 4. Conclusions

We studied the internal porous matrix of a set of rare archeological finds—ceramic vessels from the Shubarat-1 archeological site. These vessels were produced by ancient Saka tribes, who lived long ago in the territory of modern Kazakhstan. Neutron tomography was used to better understand the internal structure of these vessels. Analysis of the reconstructed three-dimensional data allowed us to identify the distribution of pores and mineral inclusions in ceramic vessels. The results showed that the distribution of pores is complex, both in terms of size and shape. This may be due to various processes, including the following: (1) spherical pores may be formed as a result of gas release during the thermal firing of ceramic vessels; and (2) elongated pores may result from the technological approaches used in ancient pottery production. In addition, large mineral inclusions were found only in two vessels, which may indicate differences in the preparation of the ceramics found at the same archeological site. The small sample size (*n* = 4) limits generalizability; larger assemblages are needed to confirm the observed patterns.

## Figures and Tables

**Table 1:** Structural characteristics of the pores and inclusions in the samples.

Sample	Total Volume, cm^3^	Porosity, %	Inclusions, %
A1	368.3(1)	0.72(1)	-
C1	341.7(1)	0.31(1)	-
K1	299.5(1)	0.78(1)	1.22(2)
Y2	396.7(2)	0.20(1)	1.68(2)

## Data Availability

The original data presented in the study are openly available. All data can be requested from the authors.
